# Resveratrol changes spermatogonial stem cells (SSCs) activity and ameliorates their loss in busulfan-induced infertile mouse

**DOI:** 10.18632/oncotarget.12990

**Published:** 2016-10-29

**Authors:** Chongyang Wu, Ying Zhang, Qiaoyan Shen, Zhe Zhou, Weishuai Liu, Jinlian Hua

**Affiliations:** ^1^ College of Veterinary Medicine, Shaanxi Centre of Stem Cells Engineering & Technology, Northwest A&F University, Yangling, Shaanxi, China; ^2^ Department of Pathology, Yangling Demonstration Zone Hospital, Yang Ling, Shaanxi Province, China

**Keywords:** resveratrol, spermatogonial stem cells, SIRT1-FOXO1, busulfan, Pathology Section

## Abstract

The decline of quantity and quality of sperm are correlated with the increasing age and some anti-cancer compounds such as busulfan. Previous studies have shown that Resveratrol (Res) inhibits tumorigenesis and metastasis of many cancers including mammary tumor, prostate and pancreatic cancers. It acts as anti-age in mouse and human, however, little is known about its protective effect on aged spermatogonial stem cells (SSCs). Here, we investigated the effects of Res *in vitro* on SSCs using C18-4 cells and *in vivo* in busulfan-induced azoospermia mice model. The results showed that Res at different concentrations had different effects on C18-4 cells. Treatment with 2 μM of Res promotes cell proliferation and inhibits apoptosis, but stimulates apoptosis with a higher concentration (20 μM) in C18-4 cells. Using busulfan-induced infertility mice model, we demonstrated that Res (30 mg/kg/d and 100 mg/kg/d) clearly ameliorated SSC loss to recover the spermatogenesis. Taken together, our data suggest that Res might be an approach for therapeutic intervention to promote SSC proliferation and cease SSC loss in azoospermia mice model induced by busulfan.

## INTRODUCTION

Spermatogonial stem cells (SSCs) are the sole adult stem cells in males which transmit genetic and epigenetic information from one generation to the next. In mice, it takes 35 days for a single SSC to generate 1024 sperms [1, 2]. The balance between self-renewal and differentiation of SSCs is regulated by a complex network, including intrinsic and extrinsic factors [2]. Any disturbance of the niche induced by aging or the detrimental environment will finally result in male infertility.

Senescence is a natural process which will cause a variety of degenerative diseases. Ample evidences prove that the decline of quantity and quality of sperm are correlated with aging [3–5]. In aged rodents, the testis is suffering from atrophy, and the spermatogenesis in it is retarded and even ceased [6, 7]. Anatomy analysis reveals that the number of functional seminiferous tubules is reducing, fibrosis is forming, and the undifferentiated spermatogonia is losing [6]. Besides the naturally increased age, there are a large number of drugs, such as busulfan, originally used in chemotherapy, can induce pre-senescence. As a nitrogen mustard alkylating agent, busulfan is widely applied in treatment of chronic myelogenous leukemia and pretreatment of hematopoietic stem cell transplantation. It kills tumor cells and destroys the immune system by attacking the structure of DNA in rapidly proliferating cells [8]. The mechanism of apoptosis induced by busulfan is similar to the aging process. Treatment with busulfan for 30 days can accelerate the senescence of female mice, down-regulating the expression of estrogen in ovary, decreasing the number of all kinds of follicles [9]. Busulfan can induce apoptosis of bone marrow hematopoietic cells of mice via causing pre-senescence in mice [10]. In 1994, Brinster etc. firstly established the azoospermia mice model by busulfan injection, which laid a foundation for the further study in spermatogenesis [11, 12].

Res is a polyphenolic phytoalexin which can mimic calorie restriction to extend the life span of *Yeast*, *C. elegans*, *Fruit flies* and mammals [13–15]. SIRT1 protein is activated to effectively alleviate the functional degeneration caused by aging and high-fat diet when Res was added to the food of mice [13, 16]. Res plays strict roles in a dose-dependent and tissue-specific manner. Also, it can be used as a chemotherapeutic drug, which can induce apoptosis of liver cancer and colon cancer cells by mitochondria, p62, GSK3β and other pathways [17–19]. Res suppresses the tumorigenesis, development and metastasis of cancers. However, little is known about the protective effects of Res on aged male SSCs. In this study, we investigated the effects of Res on SSC line C18-4 cells *in vitro* and busulfan-induced oxidative damage and apoptosis in mouse testes. The C18-4 cell line was established by stably transfecting type A spermatogonia from 6-day-old mice with the Large T antigen gene, which has phenotypic characteristics similar to primary type A spermatogonia from 6-day-old mice as evidenced [20]. Our data demonstrated that Res might be an efficient approach for therapeutic intervention to promote SSC proliferation and resume SSC loss in busulfan-induced pre-senescence mice.

## RESULTS

### Resveratrol had a dose-dependent effect on C18-4 cells

**Figure 1:**
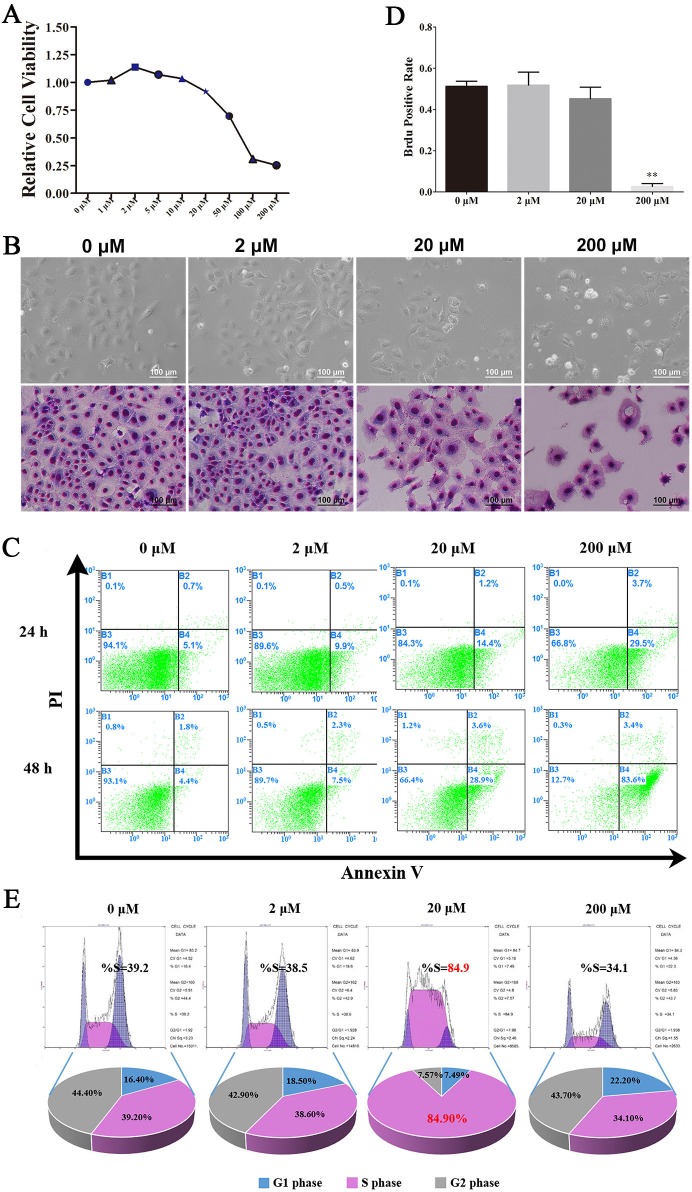
Resveratrol had a dose-dependent effects on C18-4 cells **A.** The effects of different concentration of Res on the viability of C18-4; **B.** The morphological changes of C18-4 cells after treated with Res at different concentrations, the above row is the bright field of C18-4 cells, and the low row is the Giemsa staining of C18-4 cells at different concentration of Res; **C.** The effects of Res on the apoptotic level of C18-4 cells analysed by Flow cytometry; **D.** The BrdU-positive rate of C18-4 cells after treated by Res,**, *P*<0.01; **E.** The cell cycle proportion of C18-4 cells after treated with Res analysed by Flow cytometry.

In our previously study, we first verified the identity of the C18-4 cells using various markers of germ cells and SSCs. Immunofluorescence revealed that C18-4 cells expressed PLZF, NANOS2, VASA, SSEA1 and CD49f, and negative for Stra8 (Figure S1). These showed that C18-4 cells preserved in our laboratory had the typical characteristics of the A single SSCs, which was the basis of the experiment. To further test the effects of Res on the SSCs, cell viability was detected using CCK-8 kit, and we found that low concentration Res (1 μM, 2 μM) had a promoting effect on the activity of the SSCs, however, the activity of SSCs was significantly inhibited when Res dose increased (Figure 1A). Giemsa staining showed that there was more nuclear shrinkage in C18-4 cells when treated with 200 μM Res (Figure 1B). To better understand the ability of Res in inducing apoptosis, we next performed flow cytometry assay to examine the level of the cell apoptosis after treated with different concentrations of Res. The results indicated that the apoptosis rate was highest in 200 μM Res, reaching 83.6% (Figure 1C). Consistent with this, we also found that Res in low concentration could increase the positive rate of BrdU, and after stimulated by high concentration of Res, DNA replication was inhibited (Figure 1D, S2). Furthermore, 20 μM Res could increase the cell proportion of S phase (Figure 1E). Together, these results suggested that Res provided a significant dose-dependent effect on C18-4 cells *in vitro*, with the increase of the concentration of Res, the activity of SSCs was significantly inhibited.

### Mechanisms of the effects of resveratrol on C18-4 cells

**Figure 2:**
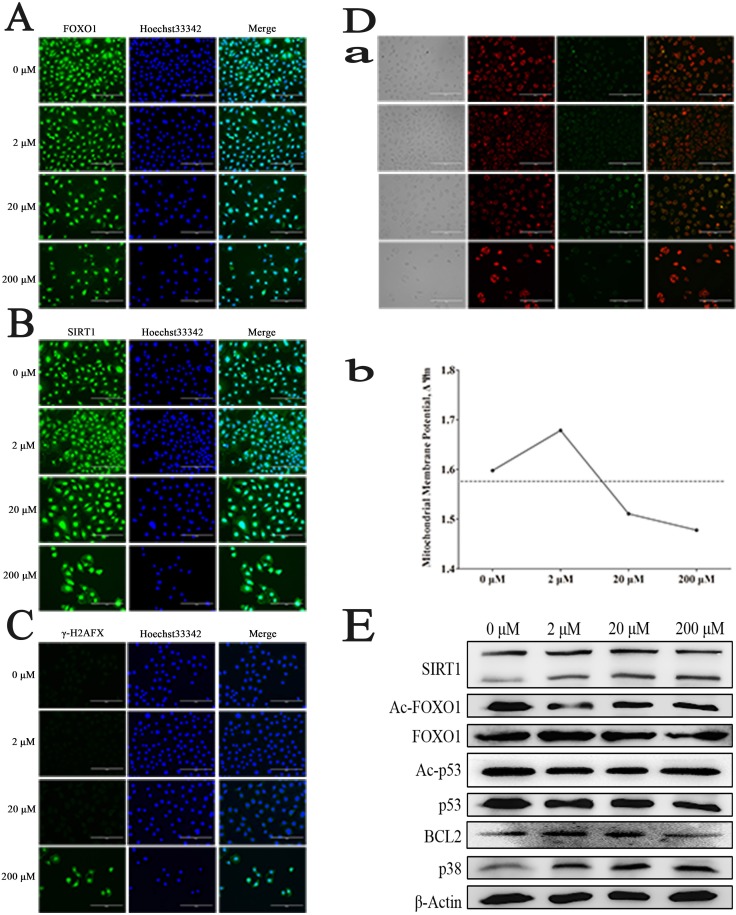
Resveratrol effects on mouse SSCs **A.** Immunofluorescence staining of FOXO1; **B.** Immunofluorescence staining of SIRT1; **C.** Immunofluorescence staining of γ-H2AFX after treated by Res; **D.** a, The changes of mitochondrial membrane potentiality (ΔΨm) of C18-4 cells after treated by Res; b, The ratio of GFP and RFP was quantitatively determined by flow cytometry; **E.** The changes of related protein level in C18-4 cells treated by Res.

It had been reported that FOXO1 expressed in SSCs, and mainly located in the nucleus, which could be the target protein of the deacetylates of SIRT1. As shown in Figure 2A, FOXO1 mainly located in the nucleus. By immunofluorescence staining, we found that SIRT1 located in the nucleus of cells in the control group, while, after treated with Res, it was also detected in cytoplasm (Figure 2B), which implied that the expression level of SIRT1 had been enhanced, meanwhile, there was the phenomenon of nuclear-cytoplasm shuttle. To further elucidate the mechanism of Res, we also used immunofluorescence staining to detect the expression of γH2AFX, which could be a signal of double DNA strand break. And the results inferred that 200 μM Res could make the double DNA strand break and further induce apoptosis (Figure 2C). Besides, we also found that low level Res protected the integrity of the mitochondrial membrane, while the middle and high concentrations of Res resulted in an opposite role, damaging the mitochondria, and further causing apoptosis, as shown in Figure 2D (a, b). Moreover, western blot analysis demonstrated that with the increase of Res dosage, the expression of Sirt1 was significantly enhanced. Res at 2 μM and 20 μM inhibited the level of Ac-p53 and Ac-foxo1, which might be due to the increased expression of Sirt1. The up-regulation of P38 and down-regulation of BCL2 indicated that increased level of Res will led to SSC apoptosis (Figure 2E). Based on these findings, we suggest that low concentration of Res allowed for mitochondrial membrane protection and SIRT1 activation, further increase the level of de-acetylation of FOXO1 in SSCs. Conversely, by down-regulating the expression of BCL2 and up-regulating the level of p38, high dose Res ultimately resulted in SSCs' apoptosis.

### Resveratrol promoted the recovery of the male germ cells after treated with busulfan

**Figure 3:**
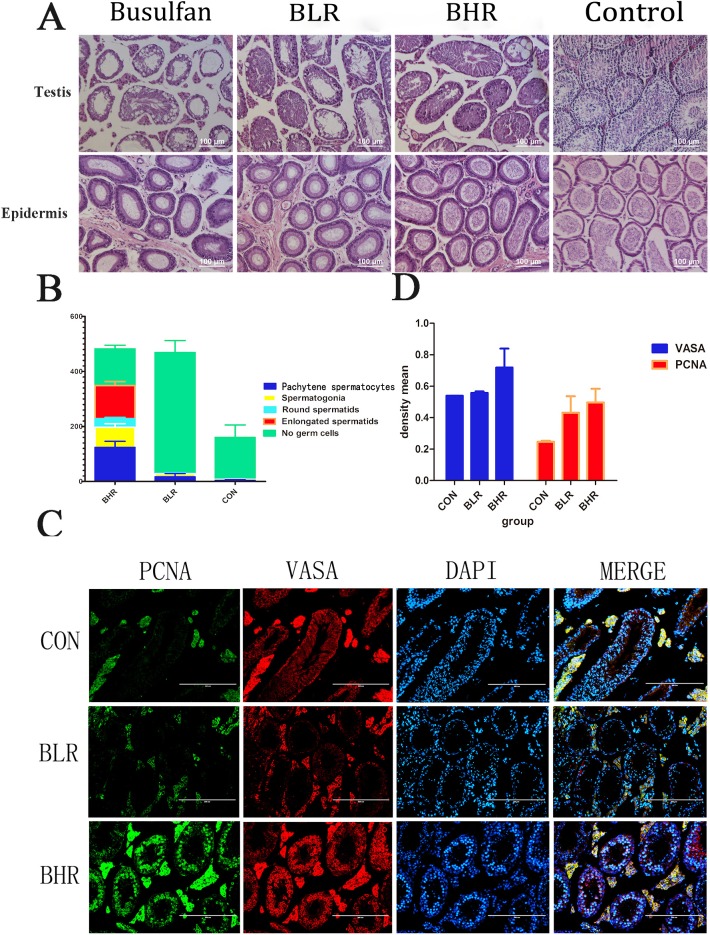
Res can promote the recovery of the SSCs after treated with busulfan **A.** H&E staining to detect the effect of Res on the SSCs of azoospermia mice; **B.** The percentage of different male germ cells in the testis of Res treated busulfan-induced infertility mice. The Y axis denotes the indicated germ cell type. The X axis denotes the different groups' analysis. The results are presented as mean±S.E.M. (*n* = 3 per group); **C.** Fluorescent immunocytochemistry analysis showed the expression level of VASA and PCNA in different groups, Scale bar = 200 μm; **D.** Image-Pro Plus analysis showed the density mean of the results of the fluorescence. (*n* = 3 per group).

It was reported that Res affected aging and recovered the tissue damage, and the dose of Res was ranged from 24 mg/kg/d to 400 mg/kg/d [13, 16]. In this study, we used 30 mg/kg/d (BLR) and 100 mg/kg/d (BHR) of Res to inject the infertility mice treated with busulfan. HE staining showed that after 2 to 3 months' Res treatment, when Res dose increased, especially in BHR group, most of the SSCs recovered, and there appeared a large number of sperms in the epididymis in the Res treated group (Figure 3A). Consistent with this, different male germ cells in BHR group had been obviously rescued (Figure 3B). Immunofluorescence analysis of testis paraffin section showed that Res significantly promoted the positive percentage of PCNA and VASA compared with control group (Figure 3C).

We next performed Image-Pro Plus analysis and demonstrated that the density mean of BHR group was the highest (Figure 3D). All these data indicated that Res in high dosage *in vivo* can significantly promote the proliferation of male germ cells, which might help therapy for patients with azoospermia or oligozoospermia.

### The potential role of Resveratrol on the busulfan-induced mice infertility

**Figure 4:**
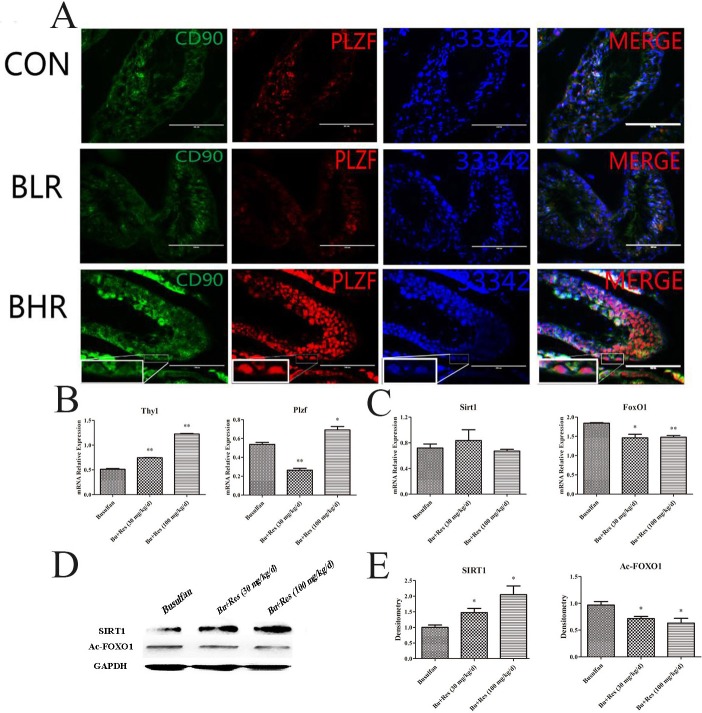
The potential role of Res function on the busulfan-induced mice infertility model **A.** Fluorescent immunocytochemistry analysis showed the expression level of PLZF and CD90 in different group; **B.** The effects of Res on the mRNA expression level of THY1 and PLZF in the busulfan-induced mice infertility model; **C.** The effects of Res on SIRT1 and FOXO1 on the mRNA expression level in the busulfan-induced mice infertility model; **D.** Western blotl revealed the change of proteins SIRT1 and Ac-FOXO1 in Res treated testis; **E.** Image-J software calculated the gray value of SIRT1 and Ac-FOXO1 (*,*P* < 0.05).

The levels of CD90 and PLZF were significantly increased in Res treatment group compared with control group (Figure 4A), which indicated that the quantity of SSCs in Res treated mice had been greatly improved, so we further detected the mRNA levels of CD90 and PLZF, and the results were consistent with the immunofluorescence analysis (Figure 4B).

**Figure 5:**
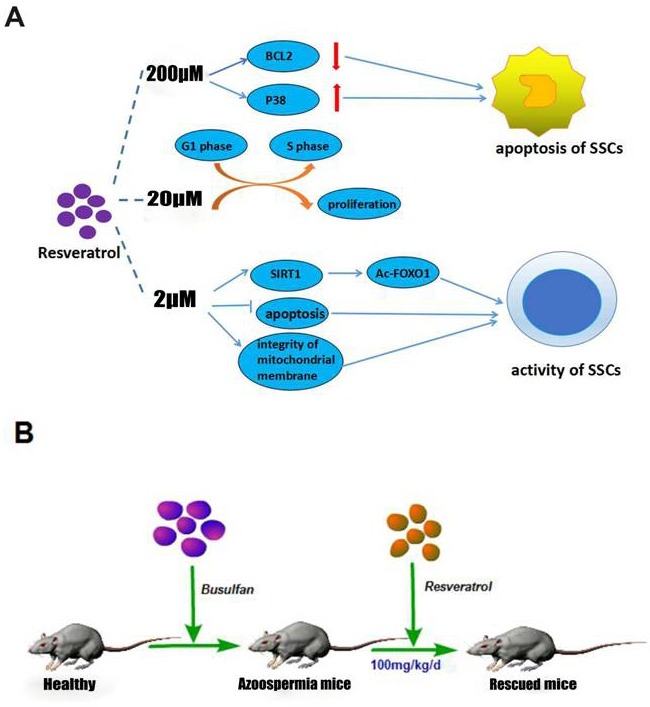
The schematic of the mechanism of Res *in vitro* and *in vivo* **A.** Different concentrations of Res had different effects on C18-4 cells; **B.** Res ameliorate SSC loss in busulfan-induced testicular damage in mice infertility model.

We next found that in mRNA level, SIRT1 had no significant change (Figure 4C), interestingly the protein level of SIRT1 had been up-regulated significantly (Figure 4D, 4E), which was consistent with that *in vitro* assay. The level of FOXO1 was also increased, furthermore, Ac-FOXO1 was decreased significantly in protein level. These data suggested that by activating the expression of SIRT1 and inhibiting the Ac-FOXO1 level, Res could restore the activity of SSCs, and further rescue the busulfan-induced mice infertility. In fact, Res in low concentration could promote the activity of the SSC' proliferation ability (Figure 5A), and in high concentration it induced apoptosis, from this we concluded that proper Res level could indeed promote the activity of SSCs, and further *in vivo* experiments proved that Res really had significant rescue effects in busulfan treated mice model (Figure 5B).

## DISCUSSION

Res' molecular structure makes it as a potential food additive or the target clinical drug [21–22]. Res can simulate calorie restriction, guaranteed the body's normal nutrition and energy consumption [16, 23–30]. It is found that Res not only inhibits the carcinogenic factors by suppressing the cancer cell proliferation, epithelial-interstitial transformation process [30–32], it also improves the level of oxidative stress in cancer cells, mitochondria damage, DNA double chain rupture and the expression of tumor suppressor gene induced apoptosis [33]. In the treatment of diabetes, Res can cause dose-dependent hypoglycemic effect and improve the insulin levels of diabetic rats induced by STZ in order to enhance the glucose uptake [34], reducing blood glucose levels by improving insulin sensitivity [35]. At the same time, high blood glucose concentration in diabetic mice and rats caused by oxidative stress of nucleic acids was also significantly suppressed under the intervention of Res [36, 37]. In addition, Res has been reported in anti-inflammatory, antibacterial, diseases such as cardiovascular diseases and neurological disorders both experiments and clinical reports [38–40].

The function of Res is highly tissue-specific and dose-dependent. In our study, we used different concentrations of Res in culturing mouse SSC line, C18-4 cells, CCK-8 and BrdU incorporation assay evidenced that different concentrations of Res played different roles on the fate of SSCs. When Res level was raised up to 200 μM, the majority of cells became apoptosis and has been floating in the medium. When we detected the cell cycle, according to the specifications only the adherent cells were collected. At this point, a few cells can still stick the plate, and most C18-4 cells had Res tolerance, therefore this period the percentage of cells in S phase was resumed. In a word, we confirm that the usage of the concentration of Res needs to be strictly determined.

Res is a polyphenol compound. More singles in the role of plants are found in its earliest [41–43], further, when it is purified as food additives or clinical drugs, our results confirmed that the mechanisms of Res are diverse and functional complex. Moreover, adding Res could also result in an up-regulating in protein SIRT1 (100 μM), adenylate cyclase (0.8 μM), and AMPK (50 μM) [44].Some other studies reported that the effects of Res related to growth factor signaling, second messenger signaling-cAMP/cGMP signal, PI3K/AKT signal, MAPKs signal, JAK/STAT signal, these signaling pathways may be reactivated and then regulate many cell activities including intracellular redox cycle and proliferation, and cell death [45].

The mechanisms of cancer cell apoptosis induced by Res are mainly through three pathways [46–50]. In our study, we believed that 2 μM Res can maintain mitochondrial membrane integrity, and provide sufficient energy for SSCs activities. In this case, Res increases SIRT1 protein level and further promotes the target by deacetylating of FOXO1. Meanwhile, the expression level of BCL2 was also increased, this is probably a consequence of inhibiting apoptosis. After adding 20 μM Res, the expression of SIRT1 is not significantly different compared with that in low Res level. However, the mitochondrial membrane potential difference is distinctly decreased, which indicated that there was a cell apoptosis trendency, also, BCL2 was compensatory increased, besides, there was the phenomenon of cell cycle arrest and p38 associated protein were correspondingly increased. We believed that high level Res affected the activity of mouse SSCs in its cell tolerance range. When the dose of Res was up to 200 μM, SIRT1 level is still high; on the contrary, the acetylation level of its target protein FOXO1 was different compared with control group, the mitochondrial integrity was suffered from great damages, BCL2 was significantly decreased, and P38 was increased enough to stimulate apoptosis. These results demonstrated that Res led to a dose-dependent effect on SSCs, therefore we concluded that proper concentration of Res can indeed promote the activity of SSCs (Figure 5A).

The busulfan induced cell damage and aging process can be resulted from the intracellular accumulation of DNA damage and gradually lead to a decline in cell viability until the final programmed cell death [51]. After 5 w busulfan treated mouse, bone marrow cells showed premature aging phenomenon and apoptosis, this process is not dependent on p53-p21 signaling pathway [52–53]. It has been confirmed that busulfan could cause reproductive toxicity in a large number of clinical trials [12]. The mouse model of busulfan-induced azoospermia has been successively laid a solid foundation for further study of spermatogenesis. We confirmed that Res (30 mg/kg/d, 100 mg/kg/d) can interfere in the busulfan-induced SSC injury. Moreover, the expression of SSC markers was increased and Sertoli cell number was relative declined, especially in 100 mg/kg/d dose, its effect is more obvious. Western blot analysis showed that SIRT1 expression in Res treatment group was significantly increased, thus de-acetylated FOXO1 proteins, which regulate apoptosis and oxidative stress related effects. Results demonstrated that appropriate concentration of Res does have significantly rescue effects in busulfan treated mice model, and even help therapy for patients with azoospermia or oligozoospermia (Figure 5B), however, whether and how Res effect by promoting SSCs regeneration so as to achieve rescuing effect still need further exploring.

Together, in this study, we used SSC cell line, C18-4 to investigate the effects of Res on male SSCs' proliferation and apoptosis, and we demonstrated that Res could be an efficient approach for therapeutic intervention to promote SSC proliferation and resume SSC loss in busulfan-induced pre-senescence mice.

## MATERIALS AND METHODS

### Cell culture

C18-4 cell line was established by stably transfecting type A spermatogonia from 6 day-old mice with the Large T antigen gene under the control of a ponasterone A-driven promoter [20, 54], and they were cultured in Dulbecco's Modified Eagle's Medium/Nutrient Mixture F12 (DMEM/F12, Invitrogen, Grand Island, NY) supplemented with 10% fetal bovine serum (FBS, Hyclone, USA), 2 mM L-glutamine (Invitrogen), and 100 unit/ml penicillin and streptomycin (Invitrogen). Resveratrol was purchased from Sigma-Aldrich (Sigma-Aldrich, MO, USA) and dissolved in dimethyl sulfoxide (DMSO) (Sigma-Aldrich) for *in vitro* study and in ethylalcohol for *in vivo* study, then diluted in ddH2O before gavage.

### Cell viability assay

C18-4 cells were cultured in 96-well plates and treated with a gradient concentration of Res (0 to 200 μM). 24 h after treatment, 10 μl of CCK-8 (Vazyme, Nanjing, China) solution per well was added and the plate was incubated for 2 h at 37oC. The absorbance of each well was measured at 450 nm by microplate spectrophotometer. The cell viability was calculated using the formulation as follows: Cell viability= (OD of treated group-OD of blank group)/(OD of control group-OD of blank group).

### Immunofluorescence and BrdU assays

C18-4 cells were cultured in 48-well plates and treated with a gradient concentration as described above for cell viability assay. The process of immunofluorescence, BrdU incorporation and staining were performed following the instruction as described in our previous studies [55].

### FACS analysis of Cell cycle and apoptosis

For all experiments, logarithmic growth phase C18-4 cells were plated in 6-well and treated with 2 μM, 20 μM and 200 μM of Res for 24 h. Then, the cells were re-suspended as single cells and washed in precooling PBS, and incubated using the Cell Cycle Kit (LianKeBiology, Hangzhou, China) with 1 ml liquid A and 10 μl liquid B for 30 min, cell cycle analysis was performed with a Beckman flow cytometer [56].

Cells for apoptosis test were resuspended gently in 500 μl PBS, and 5 μl annexin V-FITC and 5 μl propidium iodide were added to the medium and mixed gently. Cells were incubated at room temperature for10 min in the dark, then underwent FACS analysis.

### Western blotting

Proteins were extracted from C18-4 cells which were treated with various doses of Res for 24 h. Cells were collected and lysed with lysis buffer, and then the protein concentration was detected using the BCA Protein Quantification Kit (Vazyme, Piscataway, NJ, USA). After heat denaturation in 5% SDS–PAGE sample loading buffer, the protein samples were resolved by SDS-PAGE and transferred to a PVDF membrane [55–56]. The samples were probed with β-ACTIN (1:1000; Sino Biological Inc., Shanghai, China), GAPDH (1:1000; Sino Biological Inc.), p53 (1:1000; Bioss, Beijing, China), Ac-p53 (1:1000; Cell Signaling Tecnology, Inc., USA), FOXO1 (1:500; Bioworld, Nnjing, China), Ac-FOXO1 (1:500; Cell Signaling Tecnology), BCL2 (1:500; Bioss), P38 (1:500; Sangon Biotech, Shanghai, China), SIRT1 (1:1000; Bioworld) as previously described [24]. The secondary antibody was horseradish peroxidase-conjugated anti-rabbit/mouse IgG (1:1,000; Boster, Wuhan, China). Protein blots were probed with the indicated primary antibodies and appropriate secondary antibodies and protein bands were visualized using the Thermo Scientific Pierce ECL western blotting substrate, and the results were analyzed using a Tanon-410 automatic gel imaging system.

### Animal experiments and ethics statement

All experiments were performed on healthy adult male ICR mice that weighed between 25 g and 30 g. The mice were purchased from the animal center of the Fourth Military Medical University. The mice were housed in wire cages at 25°C under a 12 h light-dark cycle with 70% humidity and fed ad libitum. The maintenance of animal and the conduction of experiments were in accordance with the Guidelines for the Care and use of Laboratory Animals in Northwest A&F University. Eight-week-old ICR male mice received a single intraperitoneal injection of busulfan (30 mg/kg body weight) diluted in DMSO. After 2 weeks of busulfan pre-treatment, Res (30 mg/kg/d and 100 mg/kg/d) were injected to rescue the SSCs by gavage, each group had 7 mice. Another 2 weeks later, all the mice were executed to collect their testis.

### Statistical analysis

SPSS 17.0 software was used for statistical analysis and the results were expressed as the mean ± SD. T test was applied to evaluate the differences between groups. The level of significance was set at *P* < 0.05 and *P* < 0.01

## SUPPLEMENTARY MATERIAL


