# Supplementary material for: Resveratrol changes spermatogonial stem cells (SSCs) activity and ameliorates their loss in busulfan-induced infertile mouse
Source: Oncotarget. 2016 Oct 29;7(50):82085–96. doi: 10.18632/oncotarget.12990 (PMC5347676; doi:10.18632/oncotarget.12990)
Supplement: Supplementary file 1 [file oncotarget-07-82085-s001.pdf]

Resveratrol changes spermatogonial stem cells (SSCs) activity and ameliorates their loss in busulfan-induced infertile mouse

Supplementary Material

S1

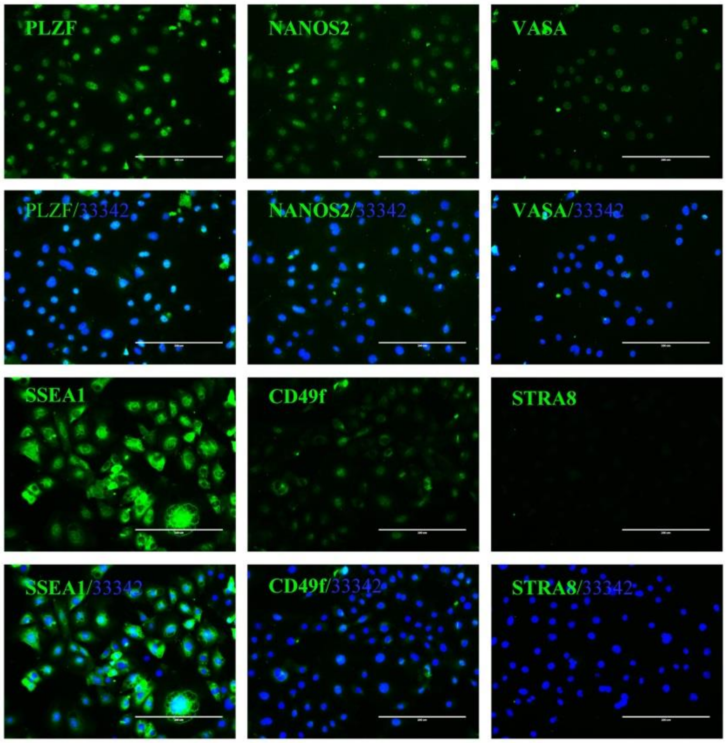

Supplement Figure 1: C18-4 cells preserved in our laboratory had the typical characteristics of the Asingle SSCs. Immunofluorescence staining of PLZF, NANOS2, VASA, SSEA1 CD49f and STRA8 (green), nucleic acids were stained with Hoechst 33342 (blue). Scale bar = 200 μm.

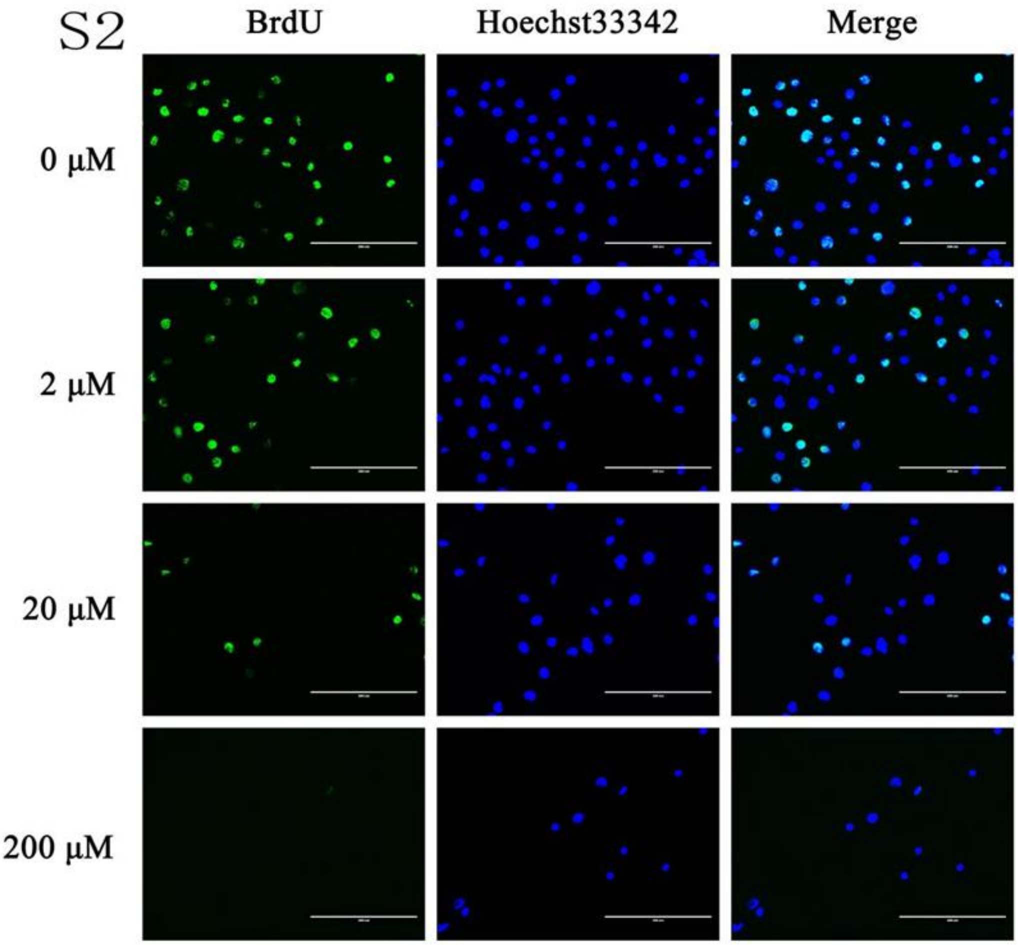

Supplement Figure S2: BrdU incorporation assay. BrdU immunofluorescence staining and percentage of BrdU-positive cells in C18-4 cell line after treated with different concentrations of Res. Scale bar = 200 μm.
